# A Comprehensive Review of the Strategies to Reduce Retinoid-Induced Skin Irritation in Topical Formulation

**DOI:** 10.1155/2024/5551774

**Published:** 2024-08-17

**Authors:** Angga Cipta Narsa, Cecep Suhandi, Janifa Afidika, Salsabil Ghaliya, Khaled M. Elamin, Nasrul Wathoni

**Affiliations:** ^1^ Department of Pharmaceutics and Pharmaceutical Technology Universitas Padjadjaran, Sumedang, Indonesia; ^2^ Department of Pharmaceutics and Pharmaceutical Technology Faculty of Pharmacy Mulawarman University, Samarinda, Indonesia; ^3^ Graduate School of Pharmaceutical Sciences Kumamoto University, Kumamoto 862-0973, Japan

## Abstract

Currently, retinoids are known for their abundant benefits to skin health, ranging from reducing signs of aging and decreasing hyperpigmentation to treating acne. However, it cannot be denied that there are various side effects associated with the use of retinoids on the skin, one of which is irritation. Several approaches can be employed to minimize the irritation caused by retinoids. This review article discusses topical retinoid formulation technology strategies to reduce skin irritation effects. The methodology used in this study is a literature review of 21 reference journals. The sources used in compiling this review are from PubMed, Scopus, ScienceDirect, and MEDLINE. The findings obtained indicate that the following methods can be used to lessen retinoid-induced irritation in topical formulations: developing drug delivery systems in the formulation, such as encapsulating retinoids, transforming retinoids into nanoparticles, forming complexes (e.g., with cyclodextrin), and binding retinoids with carriers (e.g., polymers, NLC, SLN), adding ingredients with anti-irritation activity, skin barrier improvement, and increased skin hydration to retinoid formulations (e.g., combinations of glucosamine, trehalose, ectoine, sucralfate, omega-9, and 4-t-butylcyclohexanol, addition of ethanolic bark extract of *Alstonia scholaris* R. Br).

## 1. Introduction

Retinoids are compounds that have been used topically and systemically since the 1940s to address acne problems [1]. Over the past few decades, topical retinoids have seen increasing development in their usage. Among various treatments, topical retinoids are among the most commonly used active ingredients and have been clinically tested [2]. Retinoids have been shown to successfully improve several dermatological conditions, including photoaging/rhytides, psoriasis, and acne vulgaris. They are also used off-label for conditions, such as hyperpigmentation and keratosis pilaris [3].

While retinoids offer advantages in treating several skin conditions, they can also have drawbacks, including retinoid dermatitis, characterized by redness, peeling, burning, and itching [4]. The side effects that occur are typically dose-dependent [5]. The concentration of retinoids that can cause skin irritation can vary depending on several factors, including the specific retinoid compound, the individual's skin sensitivity, and the frequency and duration of use. Generally, higher concentrations of retinoids tend to cause more skin irritation [6, 7].

Studies on the mechanism of irritation have shown that retinoid-induced irritation can be mediated by various pathways. The mechanism of retinol irritation generates broad and chronic inflammation defined by immune cell infiltration and cytokine release, as well as breakdown of the skin barrier caused by a genetic imbalance of components linked with the cornified envelope (CE) [8]. A study conducted by Kim et al. [9] demonstrated that retinoids can generate a range of proteins associated with skin irritation, such as MCP-1 and IL-8. However, the observations made so far have not provided a comprehensive explanation for the process behind retinoid-induced irritation, and as a result, definitive conclusions have yet to be established.

There are many ways to reduce skin irritation caused by retinoid products. The way a product is made affects how it works on the skin. Research is ongoing to create products that work well without causing irritation [3]. Different methods, like liposomes and solid lipid nanoparticles, are being studied to reduce irritation [10]. This review aims to explore these methods to lessen skin irritation from using retinoid products.

## 2. Method

**Figure 1:**
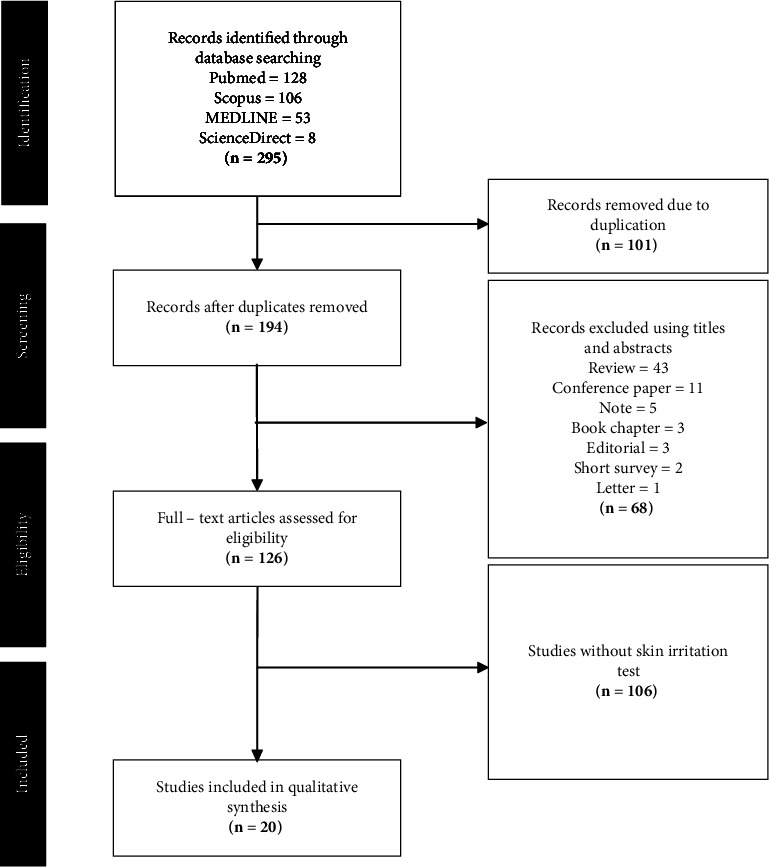
Flowchart of the search and selection process based on PRISMA guideline.

This review was conducted following the Preferred Reporting Items for Systematic Reviews and Meta-Analyses (PRISMA) guidelines, utilizing a search of articles from the PubMed, Scopus, ScienceDirect, and MEDLINE databases. The search strategy employed Boolean operators, using the syntax “Retinoid AND Irritation AND Formulation.” To enhance the comprehensiveness of the review, no restrictions were placed on the publication year during the literature search. The retrieved articles were then collated and evaluated for duplicates. Subsequently, screening of titles and abstracts of the obtained articles was performed. Further screening involved analyzing full-text articles to assess their eligibility. The eligibility of articles was determined based on inclusion criteria, including studies published in English, studies involving the use of retinoids as an active ingredient in the formulations used, and studies conducting irritation testing either in vitro, in vivo, or clinically. The exclusion criteria for this review encompassed articles, such as reviews, notes, book chapters, conference papers, short surveys, editorials, and letters. The flow of article selection leading to the identification of eligible articles is depicted in Figure 1.

## 3. Topical Formulation of Retinoid

Retinoids have been shown to successfully treat several dermatological conditions, including reducing signs of aging (wrinkles, photoaging, rhytides), decreasing hyperpigmentation, and treating acne, as well as other dermatological illnesses, such as Kaposi's sarcoma, psoriasis, and T-cell lymphoma.

### 3.1. Reducing Signs of Aging (Wrinkle, Photoaging, and Rhytides)

Vitamin A and its derivatives, particularly retinol, have demonstrated notable efficacy as pharmaceutical agents for slowing down the aging process. The efficacy of topically administered formulations containing retinoids in reducing wrinkles can be attributed to several mechanisms. These mechanisms include the stimulation of keratinocyte proliferation and collagen synthesis, enhancement of the epidermal barrier function (EGFR), prevention of collagen breakdown, reduction of transdermal water loss (TEWL), and suppression of metalloproteinase activity. Hence, the objective of this literature review is to provide a comprehensive overview of several approaches applied to mitigate the occurrence of skin irritation as an adverse effect resulting from the application of topical retinoids [11, 12]. Retinoids are thought to exert their effects at the molecular level by interacting with nuclear retinoic acid receptors, which act as transcription factors in the regulation of gene expression [13]. Retinol improves skin suppleness by eliminating damaged elastin fibers, activating fibroblasts to produce collagen fibers, increasing fibroblast number, and stimulating angiogenesis. Collagen production declines with age, leading to wrinkles and fine lines. Retinoids stimulate collagen formation, which improves skin firmness and reduces the depth of wrinkles [14, 15].

### 3.2. Decrease Hyperpigmentation

Retinoids can be used to improve melanin distribution in the skin, reducing skin discoloration and pigmentation by up to 60%. They are employed to treat pigmentation disorders, such as melasma and postinflammatory hyperpigmentation. Retinoids work by inhibiting tyrosinase and dispersing epidermal melanin. Additionally, they may cause the epidermis to shed faster, interfering with pigment transfer to keratinocytes and accelerating pigment loss. They also inhibit the activity of active melanocytes and prevent melanin transfer to epidermal cells. Long-term use of retinoids decreases melanin levels while increasing the stratum corneum thickness [15, 16].

### 3.3. Treating Acne

Topical retinoids are used for the treatment of both noninflammatory and inflammatory acne. Currently, the Food and Drug Administration (FDA) has approved three topical retinoids: adapalene, tazarotene, and tretinoin [17]. Topical retinoids play a significant role in acne treatment due to their proven efficacy in reducing visible lesions and suppressing the development of microcomedones and new lesions [18]. They function by inhibiting keratinocyte growth and promoting cellular differentiation, thereby regulating the process of desquamation [18]. In addition to their primary effects, topical retinoids have been found to inhibit the migration of leukocytes, hinder the AP-1 pathway, and disrupt various other crucial inflammatory mechanisms triggered during the development of acne [19, 20]. Furthermore, topical retinoids have been shown to improve the penetration of additional topical acne medications and facilitate the healing of postinflammatory hyperpigmentation resulting from acne [21].

### 3.4. Kaposi's Sarcoma

In the case of Kaposi's sarcoma (KSC), retinoids have been observed to indirectly inhibit its initial growth. The antiproliferative effects of retinoids can occur due to the modulation of cytokines or receptors, leading to changes in their expression. For example, retinoids can inhibit the production of oncostatin M through the autocrine IL-6 pathway, thereby exerting antiproliferative effects. Additionally, retinoids can upregulate the expression of TGF-*β*, which hinders the normal proliferation of endothelial cells. Furthermore, retinoids can influence the expression of TGF-*β* receptors, contributing to their antiproliferative properties [19, 20, 22, 23].

### 3.5. Psoriasis

Retinoids are involved in the physiological process of skin cell formation and possess a chemical composition that bears resemblance to vitamin A. The mechanism of action of retinoids involves the inhibition of keratinization, which refers to the process by which skin cells thicken due to the deposition of protein within them. Additionally, retinoids suppress excessive cell proliferation, both of which are characteristic features observed in individuals with psoriasis [11, 24]. The sole prescribed treatment for psoriasis is the topical retinoid known as tazarotene. Tazarotene undergoes hydrolysis in the tissues, resulting in the formation of tazarotenic acid. Subsequently, tazarotenic acid binds to the retinoic acid receptors. The interaction between the receptor and ligand is responsible for controlling the expression of retinoid-responsive genes, which play a role in cell proliferation and inflammation. Psoriasis, a condition characterized by increased epidermal proliferation, is distinguished by the presence of cell proliferation and inflammation as prominent symptoms [25].

### 3.6. T-Cell Lymphoma

Retinoids, as a collective class, share a common mode of action. However, it is important to note that individual retinoids possess unique structural characteristics and exhibit specific binding affinities to receptors. This molecular variability accounts for the diverse indications and results associated with different retinoids. For instance, bexarotene, a retinoid compound with specificity for the retinoid X receptor (RXR), is indicated as a suggested therapeutic option for managing cutaneous T-cell lymphoma [26]. Bexarotene binds to and activates the RXR nuclear receptors, resulting in the inhibition of the G1, G2, and M phases of the cell cycle. This leads to a reduction in cell proliferation and an increase in programmed cell death, known as apoptosis [27].

### 3.7. Skin Irritation

While retinoids have proven beneficial in dermatology, they can also cause skin irritation, with the severity often corresponding to the dosage administered. Symptoms of irritation may include erythema, pruritus, a sensation of burning, xerosis, desquamation, or the development of a condition known as “retinoid dermatitis” at the application site of the retinoid [7, 28]. Retinoids have been found to enhance the proliferation of cells in the stratum corneum and basal layer of the epidermis, promoting epidermal turnover. Consequently, the process of shedding old and damaged skin cells is accelerated. The thinning of the stratum corneum due to increased cell turnover can diminish the skin's ability to act as a protective barrier, contributing to heightened irritation and sensitivity. Research conducted in vivo and clinical settings has demonstrated that the application of topical retinoids often leads to dry skin. However, there is no empirical evidence to support the notion that sebum production is diminished as a result of this treatment. Dry skin, characterized by flaky skin, is believed to arise from the normalization of keratinocyte differentiation, proliferation, and desmosome weakening, leading to a reduction in cell adhesion [29]. Although topical retinoids have the ability to bind to retinoid receptors on sebocytes, resulting in decreased sebum production, current research does not convincingly show that topical retinoids have a sebosuppressive effect [30]. The administration of retinoids could interfere with lipid production, essential for maintaining the integrity of the skin barrier. Reduced lipid production levels diminish the capacity of lipid envelopes to function as a barrier, potentially leading to decreased defense against external irritants and increased transepidermal water loss. This heightened sensitivity may disrupt the integrity of the skin's natural barriers, increasing susceptibility to skin damage [31].

## 4. Strategies

The strategy of formulating topical retinoid technology to minimize skin irritation can be achieved through encapsulation or complex formation techniques in the drug delivery process [32, 33]. There are several methods (Table 1) for encapsulating nanoparticles. One popular strategy involves encapsulating nanoparticles with a protective layer or shell, which can be formed from polymer materials, lipid layers, or inorganic substances [52–54]. Several studies have shown that colloidal carriers such as solid lipid nanoparticles (SLN), nanostructured lipid carriers (NLC), polymer particles, liposomes, niosomes, cyclodextrins, and microemulsions can reduce retinoid irritation. Additionally, alternative methods to reduce irritation effects include the inclusion of anti-irritation substances. In this paper, we provide a comprehensive review of several studies focusing on technological advancements in topical retinoid formulations aimed at mitigating skin irritant effects.

### 4.1. Liposome

The first generation of new drug delivery vehicles, liposomes, has undergone substantial research [55]. Liposomes, consisting of bilayers referred to as unilamellar or multilamellar liposomes, are employed as carriers in various applications. These bilayers consist of concentric layers separated by aqueous compartments, created by amphipathic molecules such as phospholipids that enclose the core aqueous compartments [56]. Liposomes have the capacity to encapsulate various types of medication, allowing modifications of their appearance through lipid modification [57]. Recognized as highly efficient vehicles for drug delivery, liposomes offer exceptional biocompatibility and safety attributes. They prolong the half-life of drugs, regulate the release of drug molecules, and safeguard the encapsulated material against physiological degradation [58]. Moreover, liposomes can selectively target afflicted areas through passive and/or active delivery mechanisms, reducing the occurrence of systemic side effects, enhancing the maximum tolerated dose, and ultimately improving therapeutic outcomes [59]. The ability of liposomes to reduce the irritation caused by retinol usage stems from their capacity to protect encapsulated molecules from degradation. Additionally, the formation of liposomes enables controlled and localized release, providing sustained and targeted effects [60]. Encapsulating drugs into liposomes for topical administration leads to increased drug concentration at the targeted site of action, enhancing localized benefits and minimizing undesirable systemic side effects [61].

A study conducted by Rahman et al. [44]investigated the process of encapsulating tretinoin into liposomes generated using a modified ether injection approach. In this study, tretinoin (TRT) at a concentration of 0.025% was incorporated into a gel formulation containing 1% carbopol to evaluate its potential for causing skin irritation. Volunteers received a single application of the product (0.3 g dose) and were monitored for observable changes, namely, erythema (redness), after a 6-hour period. Outcomes were assessed using a scoring framework ranging from 4 to 0: 4 indicates severe erythema, 3 indicates moderate-to-severe erythema, 2 indicates moderate erythema, 1 indicates mild erythema, and 0 indicates the absence of erythema. The results indicated that the liposome formulation exhibited decreased erythema scores (mean score of 0.2 ± standard deviation of 0.37) compared to both the TRT 0.025% gel formulation (mean score of 1.70 ± standard deviation of 0.751) and the commercially available product (mean score of 1.40 ± standard deviation of 0.534) [41].

In another study conducted by Raza et al., the thin-film hydration process was employed to manufacture tretinoin-loaded liposomes. The liposome dispersion consisted of tretinoin, phospholipids, butylated hydroxytoluene, and cholesterol dissolved in a chloroform-methanol (2 : 1 v/v) mixture. An in vivo investigation was conducted on female Laca mice, with each group being topically treated with TRE-liposomal gel, TRE-ethosomal gel, TRE-SLN gel, and TRE-NLC gel, while a fifth group was treated with saline as a control. After 2 weeks of daily treatment (approximately 0.2 g of the formulation), any residual formulation on the skin was rinsed off. The evaluated skin area was photographed using a digital camera one hour later, and the outcomes were observed. Skin samples were taken, fixed in 10% formalin solution, stained with hematoxylin and eosin, and examined under a microscope. Images from skin irritancy tests on mice demonstrated that the developed method was well accepted on rat skin compared to other treatments on the market, as there was no visually or microscopically apparent irritation. This can be attributed to the small vesicle size and the function of liposomes in protecting the skin from direct contact with the drug trapped within the vesicles. Tretinoin (TRT) can be supplied to the epidermis gradually, reducing erythematous occurrences by being absorbed into vesicles, which also reduces the contact of the TRT carboxylic acid (−COOH) groups with the stratum corneum [38]. The small vesicle size and high TRT incorporation efficiency of this proposed mixture could help reduce skin irritation.

However, liposomes have several drawbacks, including high production costs, drug/molecule leakage, and fusion. If the temperature during manufacturing and storage is not tightly regulated, drug encapsulation may leak. Phospholipids occasionally undergo reactions resembling oxidation and hydrolysis. Lysophospholipids and peroxides are created when liposomal phospholipids are hydrolyzed and oxidized, increasing the permeability of the bilayer. Additionally, in aqueous suspensions of liposomes, aggregation, particle size increase, and drug leakage may occur at considerable rates. Liposomes are also poorly soluble and have a limited half-life. Some first-generation liposome products might not be long-term stable at room temperature [62, 63].

### 4.2. Niosome

Niosomes are nonionic surfactant-based colloidal vesicular carriers composed primarily of two types of components: nonionic surfactants and additives. The vesicular layer's development is facilitated by the nonionic surfactant, while cholesterol and charged molecules serve as additives in niosome formation [64]. Cholesterol plays multiple roles in stabilizing vesicular structures, including reducing the transition temperature from the gel phase to the liquid crystal phase and decreasing the overall hydrophilic-lipophilic balance (HLB) value of the surfactant mixture used in niosome preparation [65, 66].

In recent years, extensive research has explored the application of niosomes as drug delivery systems. Niosomes offer controlled and/or sustained release of pharmaceutical substances to targeted areas while demonstrating an extended period of stability [67]. They can encapsulate large amounts of materials in a small volume, are more effective than conventional oily formulations, and can entrap a diverse range of chemicals due to their unique structure [67–69]. Properties such as shape, fluidity, and size can be easily adjusted by modifying their structural composition and synthesis technique [70]. Previous studies have shown that incorporating niosomes into gel matrices can improve drug absorption and reduce skin irritation due to their particle size, surface composition, and characteristics [71]. Niosomes, as second-generation vesicular carriers, have gained attention as suitable replacements for liposomes due to their enhanced chemical stability, superior drug encapsulation capabilities, inherent ability to enhance skin penetration, and cost-effectiveness [72, 73].

Rahman et al. investigated the in vivo irritant effects of topical proniosomal formulations containing tretinoin. Proniosomes can be hydrated immediately before use to produce niosomes. The proposed formula, N8G, consisted of protonosomes synthesized with 0.025% TRT and a 3 : 1 molar ratio of cholesterol to Span 60. Skin irritation tests were performed by comparing it to a commercial product, revealing minor erythema skin irritation test results for the proniosomal TRT gel compared to other treatments [44].

Kim et al. conducted an investigation using the lipid hydration method to generate niosomes. A cohort of 23 healthy individuals received daily administration of a 0.05% RA-N nanoemulsion blend for 4 weeks. The Visioscan® method was employed to compute the average desquamation index, which showed a significant reduction in desquamation, indicating the potential for skin tolerance without causing irritation [43].

Despite the mentioned advantages, aqueous solutions of niosomes have a short shelf life due to fusion, aggregation, drug leakage, and hydrolysis [74]. Stability is affected by factors, such as surfactant type, storage temperature, interfacial polymerization of surfactant monomers, detergents, membrane-spanning lipids, nature of encapsulated drug, and inclusion of charged molecules [3, 4].

Niosomes and liposomes have similar applications in drug delivery but differ chemically in their structure units. Niosomes are made of nonionic surfactants, whereas liposomes are made of phospholipids [75]. They function as amphiphilic vesicles, carrying out similar tasks and sharing morphological traits. Both can be used for targeted and sustained drug delivery systems [76]. Studies have shown that niosomes and liposomes both have comparable functions in vivo [77]. While having similar characteristics to liposomes, niosomes offer several advantages, including intrinsic skin penetration-enhancing qualities, increased chemical stability, and lower costs [67].

### 4.3. Solid Lipid Nanoparticle (SLN)

Solid lipid nanoparticles (SLNs) are lipid-based nanoparticles characterized by a solid core, capable of encapsulating both hydrophilic and hydrophobic pharmaceutical compounds [78]. They offer advantages such as enhanced physical stability, controlled release properties, and ease of preparation, making them safer and more cost-effective than polymer nanoparticles [35, 79, 80]. SLNs have shown potential as a drug delivery system for topical applications due to their ability to enhance drug permeation into the skin and reduce potential toxicity and irritation upon dermal application [81, 82].

Several studies have demonstrated that SLNs can reduce or even eliminate skin irritation caused by retinoids. Rodrigues et al. used an ion-pairing approach to create SLNs for encapsulating adapalene. In vivo irritation tests on female rats over a 7-day period showed significantly lower erythema scores for SLN-SA-AD gel compared to commercially available AD gel (*p* < 0.05) [34].

Shields et al. employed high-pressure homogenization to generate dispersions of SLNs, which were incorporated into polymer gels. Skin irritation tests on albino rabbits showed no erythema or edema, indicating that the formulated substances did not induce skin irritation [35].

Ahmad Nasrollahi et al. examined the creation of SLNs using a hot-melt homogenization approach combined with ultrasound emulsification. Female rats treated with SLNs containing RA-STE showed significantly reduced irritation indices compared to those treated with a commercially available RA cream (*p* < 0.05) [42].

Despite the advantages of SLNs, such as protection from adverse environmental conditions, ease of large-scale synthesis, biocompatibility, and biodegradability, they also have limitations. These include low drug loading efficiency, potential drug expulsion due to crystallization during storage, and initial burst release. The propensity for drugs to expel from SLNs during storage due to crystallization restricts drug loading. To overcome these limitations, nanostructured lipid carriers (NLCs) can be employed to prevent crystallization [83–85].

### 4.4. Nanostructured Lipid Carriers (NLCs)

Nanostructured lipid carriers (NLCs) are lipid-based nanoparticles composed of a mixture of solid and liquid lipids, resulting in an imperfect crystal structure [86]. NLCs offer advantages over SLNs, including higher drug loading capacity, minimized drug expulsion due to lipid crystallization, increased drug solubility in lipid matrix, and more controllable release profiles [87, 88]. The addition of oil in NLCs prevents lipid recrystallization, resulting in a more thermodynamically stable system with improved release properties [89–91].

NLCs are considered an enhanced version of SLNs, featuring optimized core composition, increased drug loading capacity, enhanced stability, and the ability to function at reduced temperatures [87, 91–93]. Despite being solid at physiological body temperature, NLCs have a lower melting point than SLNs, providing more space for drug dissolution and higher loading capacity [87, 88]. NLCs are less susceptible to gelation during preparation and storage, facilitating nanoparticle separation from the medium and dosage form preparation for parenteral administration [87, 88].

Autoclaving can be employed to sterilize NLC solutions, but this method requires strong dilution of the particle dispersion and elimination of organic solvent residues, posing challenges for industrial application [94]. The use of retinoid active components in NLCs has shown enhanced skin penetration due to the lipid composition of the nanocarrier, reducing the likelihood of skin irritation and photosensitization. Clinical studies have demonstrated the nonirritating nature of NLC formulations containing retinyl palmitate and tretinoin [37–40].

Castro et al. reported that NLCs prepared using ultrasonic homogenization and high-pressure techniques demonstrated skin tolerance in clinical studies [40]. Rahman et al. found that NLCs loaded with retinyl palmitate minimized irritant effects, with in vitro tests showing cell survival above 90% for all NLC formulations [39]. Pople and Singh observed no irritation with NLC-based tretinoin gel compared to a commercially available gel, which induced significant irritation within three days [37]. Raza et al. also demonstrated superior skin tolerance of NLCs compared to a commercially available product in animal studies [38].

### 4.5. Polymer

Polymer drug delivery systems are defined as formulations or devices that facilitate the entry of therapeutic substances into the body [95]. Drug carriers utilizing polymers are available in various forms, including nanoparticles, micelles, dendrimers, and hydrogels. These carriers consist of either natural or synthetic polymers, such as poly-D, L-lactide coglycolide (PLGA) or polylactide (PLA) [96, 97]. Loading drugs into these carriers can be achieved through covalent bonding, surface adsorption, or trapping within the polymer matrix [98]. These systems can enhance the safety and effectiveness of drugs by controlling the rate, timing, and location of drug release within the body. Additionally, they can protect and facilitate the release of encapsulated drugs by preventing physical and chemical degradation [95]. Drug delivery carriers offer significant capabilities, and the use of smart polymers can improve drug transport and reduce the adverse effects associated with these therapeutic agents, thereby enhancing therapeutic effectiveness [99].

The solubility of poorly water-soluble medicines can be improved by polymers, increasing their bioavailability and therapeutic impacts. Polymers have significantly contributed to the advancement of drug delivery technology by enabling cyclic dosage, adjustable release of both hydrophilic and hydrophobic medicines, and regulated release of therapeutic agents in steady doses over extended durations [100, 101]. Using a polymer as an inert carrier offers many benefits. For example, polymers can improve the pharmacodynamic and pharmacokinetic properties of biopharmaceuticals by lengthening their plasma half-lives, lowering their immunogenicity, boosting their stability, increasing the solubility of low-molecular-weight drugs, and potentially enabling targeted drug delivery [102]. Polymeric nanoparticles are excellent options for drug delivery. However, toxicity and a higher likelihood of particle aggregation are drawbacks of polymeric nanoparticles (NPs). Although polymeric nanocarriers are being tested in several clinical trials, only a small number of polymeric nanomedicines have received FDA approval and are being utilized in clinical settings [103]. The effectiveness of these nanopharmaceuticals is limited by safety issues, toxicity risks, insufficient biocompatibility, and physiological limitations [104]. Drawbacks of these nanoparticles include harmful monomer aggregation, residual material attached to them, and toxic disintegration [105].

#### 4.5.1. Polyolprepolymer-2

Polyolprepolymer-2 (PP-2) is a composite material consisting of hydroxyl-terminated polyurethane polyol with a high molecular weight and propylene glycol. Incorporating polyolprepolymer-2 into topical formulations alters the transdermal delivery of tretinoin, thereby reducing tretinoin-induced irritation [106]. Niemiec et al. [107] suggested that adding polyolprepolymer-2 to the formulation can hinder the migration of tretinoin into the deeper layers of the epidermis, consequently decreasing irritation. Quigley and Bucks [47] conducted a study where they formulated tretinoin into cream and gel forms using polyolprepolymer-2. The study demonstrated the effectiveness of polyolprepolymer-2 in minimizing the adverse effects of tretinoin-induced irritation. Three different test substances were used in patch tests on the shaved dorsal skin of guinea pigs: a 0.025% tretinoin gel containing polyolprepolymer-2, a commercially available 0.025% tretinoin gel, and a 0.025% tretinoin gel without polyolprepolymer-2. Preliminary findings indicated that the formulation with polyolprepolymer-2 resulted in significantly less pain compared to the other formulations. While the irritation scores for the 0.025% tretinoin cream with polyolprepolymer-2 suggested reduced irritation risk, no significant difference was observed compared to the 0.025% cream [47].

Luckya et al. also investigated the formulation of tretinoin with polyolprepolymer-2. Patch tests were conducted on human skin to evaluate irritation levels. A comparative analysis was performed between a tretinoin gel containing polyolprepolymer-2 at a concentration of 0.025% and an over-the-counter tretinoin gel. The results showed that tretinoin combined with polyolprepolymer-2 led to a statistically significant reduction in facial peeling compared to using the commercially available tretinoin gel [48].

#### 4.5.2. Conjugated Polymer

Comparing this drug-polymer conjugate to standard small-molecule therapy reveals several significant advantages. First, conjugating a drug with a water-soluble polymer can greatly enhance its solubility in aqueous solutions [108]. The drug-polymer conjugate offers potential for controlled drug delivery, releasing the drug in a controlled manner over a specific period. This allows for precise control of the rate and duration of drug administration to achieve the desired therapeutic concentration effectively [109]. Drug-polymer conjugates, composed of polymers, generally exhibit extended half-lives, improved stability, enhanced water solubility, reduced immunogenicity and antigenicity, and increased tissue or cell specificity [110]. Both polymeric and molecular prodrugs use polymers as carriers for proteins, targeting moieties, and imaging agents. Drug-polymer conjugates can be viewed as drug delivery systems that achieve their therapeutic efficacy by gradually releasing small drug molecules from the polymer chain [111].

Research by Castleberry et al. demonstrated that the synthesis of polymer conjugates involves covalently binding the drug to a hydrophilic polymer, polyvinyl alcohol (PVA), through ester bonds. This process produces an amphiphilic nanomaterial soluble in water, which can be hydrolytically degraded. The PVA-bound all-trans-retinoic acid acts as a prodrug, facilitating its accumulation in the skin and enabling controlled and sustained release of active all-trans-retinoic acid. In vivo irritation experiments were conducted on laboratory animals, specifically rats. Rats were administered two distinct treatments: PATRA, ATRA, PVA, and PBS, applied to a 1 cm^2^ area on their dorsal surface. Irritation assessment involved digital imaging to examine the application areas of ATRA, PATRA, or the control solution for up to five days postadministration. Inflammation was further examined using histological analysis. Each rat received one of the four test chemicals applied at two separate locations. Evaluation of inflammation and skin changes following all-trans-retinoic acid (ATRA) application was conducted using digital imaging and hematoxylin and eosin (H&E) histology. The in vivo assessment of irritation caused by the PVA-ATRA polymer-drug combination, PATRA, demonstrated a significant reduction in inflammation and irritation [46].

### 4.6. Cyclodextrins

Cyclodextrins (CDs) are cyclic oligosaccharides composed of at least six, seven, or eight glucose units connected by *α*-1,4 glycosidic linkages. Cyclodextrin glycosyltransferase (CGT) is an enzyme commonly found in *Bacillus macerans*, *Klebsiella pneumoniae*, and Alkalophilic bacteria strain number 38. This enzyme primarily facilitates starch hydrolysis, leading to the production of cyclodextrins [49]. Complexation of a substance with cyclodextrin can improve its delivery characteristics without affecting its activity, as complexation is a rapid, reversible, and dynamic process [112]. The formation of inclusion complexes between drugs and cyclodextrin enhances the physicochemical and biological properties of poorly soluble drugs and encapsulates lipophilic drugs within the cyclodextrin cavity [113]. CDs can modulate the rheological properties of creams and gels and reduce skin irritation caused by active ingredients [114]. CDs and their derivatives offer significant advantages in transdermal drug delivery, including enhanced drug solubility and stability, improved transdermal absorption, sustained drug release, reduced side effects, and facilitation of both local and systemic administration [115, 116].

CDs have been shown to improve the stability, tolerance, apparent solubility, and organoleptic properties of active substances, as well as their controlled release in the skin [114]. While CDs are too large to permeate the stratum corneum (SC), they can act as skin permeation enhancers by increasing the perceived solubility of active substances and providing an in situ reservoir effect [117]. Using CDs as skin carriers can increase the stability of active ingredients in the formulation and at the target site, reduce irritation caused by active ingredients, and allow for controlled release [118]. CDs can enhance the bioavailability of complex compounds by improving their solubility, stability, penetration, and retention, and they exhibit superior biocompatibility, which limits toxicity associated with active ingredients [119].

A study by Anadolu et al. found that beta-cyclodextrin (−CD) complexes reduced itching caused by retinoids. This study compared the effects of various topical applications on acne vulgaris patients, including a cyclodextrin complex hydrogel formulation (0.025%), cyclodextrin complex in a moisturizer base (0.025%), hydrogel base, moisturizer base, and a commercial retinoic acid gel (0.05%), through in vivo irritation testing. Only one patient discontinued treatment due to significant irritation, and none of the patients treated with the cyclodextrin complex experienced substantial local irritation compared to currently available retinoic acid formulations [49].

Another study by Kaur et al. focused on evaluating the irritating effects of isotretinoin solid inclusion complexes produced through freeze-drying. Rabbits were used as experimental subjects, and their dorsal fur was removed 24 hours prior to the experiment. The results showed that the isotretinoin elastic liposomal formulation exhibited significantly reduced skin irritation potency compared to the unencapsulated medication [50].

Despite the advantages of topical formulations, there are also limitations [120]. Cyclodextrins can only improve the topical delivery of drugs in the presence of water. In aqueous vehicle systems, cyclodextrins solubilize lipophilic, water-insoluble drugs and deliver them to the barrier surface. Drug molecules then partition from the cyclodextrin cavity into the lipophilic barrier at the surface. As a result, drug administration from aqueous cyclodextrin solutions is both diffusion and membrane-regulated. Only trace amounts of hydrated cyclodextrin molecules and drug/cyclodextrin complexes can pass through intact skin [121]. In some cases, the complexation efficiency is low, requiring relatively large volumes of cyclodextrin to complex modest amounts of a particular medication.

The availability of products containing CDs is also limited by high implementation costs for practically all CD formulations and issues with the quality and legal status of derivatives. CDs cannot permeate biological membranes during topical administration under normal conditions, but they can significantly alter drug bioavailability. Unfortunately, CDs can interact with formulation components and produce physicochemical stability issues; selecting the correct carrier appears to be a challenge. Thus, it is easier to create solutions or suspensions than to formulate complex semisolid carriers, and this tendency is reflected in the number of products available. Despite these challenges, a few complex formulations with properties modified by CD addition exist, such as liposomes, fabrics, hydrogels, microspheres, and emulsions. While these are promising forms, their practical application will likely be limited to a select few items in the future [122].

### 4.7. Nanoemulsion

Nanoemulsions are transparent systems typically composed of particles ranging from 20 to 500 nm in diameter [123]. The Brownian motion of nanoemulsion droplets provides sufficient force to counteract physical destabilization processes, such as gravitational separation, flocculation, and coalescence due to their small droplet size [124]. Nanoemulsions are multiphase colloidal dispersions that do not form spontaneously [125]. According to Kale et al., a nanoemulsion is a dispersion of water, oil, and surfactant that generates nanoscale particles through mechanical forces, forming a stable isotropic and thermodynamic system with a distributed droplet diameter [126]. The small droplet size of nanoemulsions allows for uniform deposition and penetration of active compounds across the skin's surface [127]. The increased effectiveness of nanoemulsions in facilitating material penetration can be attributed to their large surface area and lower interfacial tension within the emulsion system, requiring a surfactant concentration of approximately 3–10% during production [128]. Nanoemulsions are recognized as superior nanocarriers compared to other emulsion systems due to their stability against temperature fluctuations and dilution [129]. Their long-term stability and ability to prevent drug degradation make them an optimal drug delivery system [130]. Nanoemulsions are efficient carriers with a higher surface area and free energy, are nontoxic and nonirritating, and can be easily applied to mucous membranes and skin in various formulations, such as creams, liquids, foams, and sprays [131]. They have been shown to be excellent carriers for the optimal dispersion of active substances in specific skin layers or cells [132]. Using high-energy machinery to produce nanoemulsions can help avoid the incorporation of potentially irritating surfactants or active substances [133].

A study by Prasad et al. demonstrated that a nanoemulsion formulation containing 0.1% w/w adapalene and 1% w/w clindamycin phosphate exhibited reduced irritant properties. A comparative analysis evaluated the efficacy of a gel formulation containing a combination of 0.1% adapalene and 1% clindamycin versus a standard gel formulation in individuals with facial acne vulgaris. The results indicated a significant decrease in the occurrence and severity of adverse effects, such as local irritation (4.2% vs. 19.8%; *P* < 0.05) and erythema (0.8% vs. 9.9%; *P* < 0.05), when comparing the nanoemulsion-containing formulation to the one without [45].

Despite their benefits, nanoemulsions have drawbacks. High concentrations of surfactant and cosurfactant are required to stabilize the nanodroplets during emulsion formation, as well as high concentrations of emulsifiers. They typically have limited ability to solubilize highly melting compounds, and their stability is affected by temperature and pH, making them less stable under certain conditions [134].

### 4.8. Glucosamine, Trehalose, Ectoine, Sucralfate, Omega-9, and 4-t-Butyl Cyclohexanol Addition

To reduce the risk of irritation, it is essential to take preventative measures. Kang et al. conducted a study to examine the efficacy of a product called AF (anti-irritant formula) in reducing retinol-induced irritation. The formulation consists of sucralose, trehalose, ectoine, glucosamine, omega-9, and 4-t-butyl cyclohexanol combined with retinol. After a genetic analysis aimed at identifying factors linked to retinol-induced irritation, retinol synthesis was performed using chemical compounds capable of regulating the molecular mechanisms behind retinol-induced pathogenesis in vitro [4].

Previous studies have shown that retinoids can alter both the structure and function of the skin barrier. Applying retinoic acid to skin tissue, in both ex vivo and in vivo settings, led to an apparent increase in transepidermal water loss (TEWL), a known manifestation of compromised skin barrier function. The efficacy of sucralfate, glucosamine, and trehalose in mitigating retinol-induced disruptions to the epidermal barrier was demonstrated by their modulation of COL6A2, AQP3, and FLG gene expression. Additionally, glucosamine and ectoine administration resulted in a decrease in IL-4R overexpression generated by mast cell activation. The exact impact of ectoine, a compound found in certain halophilic bacteria, on IL-4R upregulation remains unclear. However, previous studies have shown its ability to modulate several cytokines and chemokines during inflammatory conditions. TRPV1 activation has been associated with retinol-induced irritation in an in vitro model. Inhibiting TRPV1 with 4-t-butyl cyclohexanol and omega-9 oleic acid reduced neurogenic inflammation. The research demonstrated that retinoic acid and retinol facilitate the activation of the EGFR pathway, a crucial component in the functioning of the epidermal barrier. Additionally, excessive AQP3 production induced by retinol can be reduced with trehalose [4, 135, 136]. The use of trehalose in topical formulations can reduce side effects, such as skin irritation [137].

Further investigations have substantiated the efficacy of AF components in reducing skin irritation. As reported by Bissett et al., glucosamine enhances hyaluronic acid production, accelerates wound healing, improves skin hydration, and reduces the appearance of creases. Ectoine, a cyclic amino acid produced by extremophilic bacteria, improves cell membrane hydration by retaining water on the surface, thereby reducing TEWL. Omega-9 also aids in reducing TEWL, rebuilding the damaged epidermal lipid barrier, and stabilizing skin metabolism [138–144].

Kang et al. conducted an in vivo irritation test on seven participants (three men and four women), comparing a control cream (retinol 5000 IU) with an AF-based cream with retinol 5000 IU. After three days of treatment, participants discontinued product use for four days before measuring TEWL and skin redness. The combination of ingredients significantly reduced retinol-induced irritation in the human trials. The study successfully demonstrated AF's efficacy in reducing retinol-induced irritation, specifically reducing desquamation by 66.67%, burning sensation by 68.42%, and stinging sensation by 68.97%, compared to the control retinol cream [4].

### 4.9. Plant Extract Addition

The increasing popularity of plant-derived natural medicines can be attributed to their reduced incidence of adverse effects, improved patient tolerance, and cost-effectiveness. Additionally, herbal medicines offer viable solutions for various disorders that often lack effective conventional treatments and long-term solutions [145]. Several studies have investigated the impact of botanical extracts on skin health, including improving hydration, strengthening the skin barrier, and reducing irritation.

A study by Lee et al. explored the potential of the ethanol extract of *Alstonia scholaris* R. Br. bark in treating cutaneous irritation induced by retinoids [51]. The study demonstrated the inhibitory effects of the ethanol extract on inflammation induced by all-trans-retinoic acid in human keratinocyte cells (HaCat). The extract, known as ASE, contains a diverse range of alkaloids, flavonoids, and terpenoids, including alstonidine, alstonine, chlorogenic acid, ditain, echitamine, and echitenin [146, 147]. Two significant ASE compounds known for their anti-inflammatory properties are echitamine and loganin [148]. The study aimed to evaluate the efficacy of *Alstonia scholaris* extract in alleviating cutaneous irritation through cumulative irritation experiments on human participants. The results showed that the emulsion containing *Alstonia scholaris* extract had a lower irritation score (1.95) compared to the emulsion without the extract (5.33) [9]. This suggests that the extract, particularly echitamine and loganin, has potential as a counterirritant agent in reducing negative skin reactions caused by retinoid therapy.

Skin hydration is crucial for maintaining skin homeostasis. Alterations in hydration levels can significantly impact the skin's features and functions [149]. Dry skin is associated with increased irritation, flakiness, mechanical failure, and other issues. Maintaining adequate moisture levels is challenging, especially in individuals with sensitive skin [150]. Compromised barrier function leads to increased transepidermal water loss (TEWL), which can result in various dermatological conditions, including dry skin (xerosis), acne vulgaris, atopic dermatitis, retinoid-induced dermatitis, rosacea, and psoriasis [151].

A study by Ratz-Lyko et al. demonstrated the significant impact of a *Centella asiatica* extract-based emulsion on skin hydration and epidermal barrier function, particularly in terms of tightness [152]. The presence of carbohydrates and naturally occurring antioxidants, such as saponins, flavonoids, and phenolic compounds, is believed to contribute to these effects [153]. The emulsion containing a 5% concentration of *Centella asiatica* extract showed significant improvements in skin hydration and epidermal barrier function in in vivo experiments [154]. Based on these findings, *Centella asiatica* may warrant further research for its role in reducing retinoid-induced skin irritation.

### 4.10. Others

#### 4.10.1. Encapsulation and Controlled Release Silicone Particle

The study conducted by Shields et al. focused on the development of silicone particles designed to encapsulate, protect, and control the release of retinol and other hydrophobic chemicals. The particles were synthesized through the sol-gel polymerization process using silane monomers capable of efficiently encapsulating retinol (>85%), providing protection against degradation. This encapsulation led to a significantly prolonged half-life of retinol, approximately nine times longer than that of nonencapsulated retinol. Additionally, the encapsulated retinol was released gradually over several hours. To assess the efficacy of the developed system in reducing retinoid-induced irritation, an in vivo experiment was conducted. This experiment involved applying 0.2 mL of the test formulation to an occlusive hypoallergenic patch, which was then placed on the infrascapular region of the test subject's back. The patch was removed within a 24-hour period, followed by a comprehensive assessment of the skin's response. The results from the irritation test indicated that the formulation using silicone particles exhibited lower irritation levels than the formulation incorporating microsponge particles, despite both formulations having an equivalent concentration of retinol. Specifically, the formulation with 0.2% loaded retinol using silicone particles had a cumulative irritation score of 296.0 ± 37.6, while the microsponge particles with 2% retinol generated a cumulative score of 383.0 ± 29.55. These findings suggest that a reduced rate of retinol release is associated with decreased skin irritation in human subjects [35].

#### 4.10.2. Encapsulated Nanoparticle

The study conducted by Bai et al. focused on formulating Gravi-A nanoparticles through the encapsulation of retinyl propionate (RP) and hydroxypinacolone retinoate (HPR) using the high-pressure homogenization technique. The efficacy of these nanoparticles in treating wrinkles was observed to demonstrate notable stability and minimal discomfort. To evaluate the reduction in irritation caused by retinol, an in vivo irritation test was conducted on rabbits. The test substance, approximately 0.5 g, was applied to the skin once daily for a duration of fourteen days. The mean daily irritation score per animal was determined to be 0.68, indicating a grade of mild irritation based on the skin irritation intensity categorization. The composite of HPR and RP nanoparticles, referred to as Gravi-A, demonstrated favorable stability and a notable capability for drug loading. Compared to free Gravi-A, the encapsulated form of Gravi-A exhibited a reduced IC50 value in the fibroblast model, along with enhanced skin retention and deeper penetration. These findings suggest that encapsulation of Gravi-A leads to improved safety [8].

#### 4.10.3. Invasome

Invasomes are a novel class of vesicular drug delivery systems that have gained prominence in the field of cutaneous drug administration across various therapeutic applications. Considered the next generation of liposomes, invasomes are more elastic, flexible, and permeable through the skin than liposomes and ethosomes [155]. The structures of these vesicles comprise phospholipids, ethanol, and terpenes [156]. Phospholipid vesicles enable targeted skin delivery of lipophilic drugs, increased penetration of hydrophilic medications, and reduced drug irritation [157]. A study by Jain et al. utilized carbomer to successfully create a berberine-loaded invasome gel. Skin irritation tests revealed that the manufactured invasomal gel is nonirritating and stable at freezing temperatures. Entrapment of berberine in an invasomal gel formulation enhances its biological activity, likely due to improved skin penetration. Additionally, the berberine-loaded invasomal gel releases the medication gradually over time, resulting in a sustained analgesic effect [158].

#### 4.10.4. Film-Forming Sprays

Film-forming spray (FFS) is a drug delivery system that, when sprayed, forms a film upon reaching the target therapeutic site, using polymers as a matrix for film formation [159–161]. FFS offers significant advantages over traditional topical preparations. It provides consistent medication distribution and dosage, enhanced bioavailability, reduced incidence of irritation, sustained drug release, and accelerated wound healing through moisture control [162]. After application to the skin, the composition of the film-producing system undergoes significant changes due to the evaporation of volatile components, resulting in a residual film on the skin surface. This process increases the drug concentration, potentially leading to supersaturation levels on the skin's surface. Supersaturation enhances drug flux through the skin by increasing the formulation's thermodynamic activity without altering the skin barrier, thereby minimizing side effects or irritation [163, 164].

Lu et al. investigated a metered dosage transdermal spray (MDTS) formulation for dexketoprofen (DE) transdermal administration using rats as an animal model to study skin irritability. The findings suggest that MDTS could be a promising and innovative therapeutic technology for transdermal drug administration, reducing skin irritation. Based on the current research with this animal model, it appears that the application does not irritate the skin. Initial skin irritation tests with the optimized formulations on rat skin did not show any redness or swelling. Furthermore, primary skin irritation studies of the improved formulations on rats revealed no evident erythema or edema [165].

#### 4.10.5. Ionto-Sonophoresis

Iontophoresis is a physical technique that uses a low-intensity electrical current to transport molecules through the skin [166]. This technique has various biomedical applications, particularly in facilitating the delivery of drugs, including hydrophilic and/or large-sized molecules, to targeted areas [167]. While iontophoresis offers several advantages, its use can be limited by the potential for skin irritation. However, employing various enhancement strategies may reduce the need for high-intensity currents to achieve therapeutically effective delivery volumes, thereby mitigating skin irritation concerns [166].

Several studies have indicated that the combination of sonophoresis and iontophoresis does not induce skin irritation [168–170]. Using a Franz diffusion cell, the synergistic effect of these techniques in the transdermal delivery of various cosmeceutical drugs has been demonstrated. This combined approach is beneficial as it reduces energy density and, consequently, skin irritation [168]. Long Le et al. demonstrated how sonophoresis and iontophoresis could enhance heparin's transdermal penetration. When these two techniques are combined, a lower voltage or current suffices to achieve the desired transdermal enhancement, thereby reducing skin irritation [171]. One proposed hypothesis suggests that the enhanced effect is primarily due to increased molecule diffusivity from sonophoresis in the stratum corneum, coupled with the electro-osmotic flow of water induced by iontophoresis [172]. This highlights the potential of iontophoresis and sonophoresis in minimizing skin irritation associated with retinoid administration.

#### 4.10.6. Hydrophase Base

Sawleshwarkar et al. investigated the efficacy and tolerability of 4% benzoyl peroxide cream formulated in a hydrophase base (Brevoxyl) for treating acne vulgaris. The study assessed the effectiveness and acceptability of Brevoxyl®, a 4% BP (benzoyl peroxide) cream in a hydrophase base previously available exclusively by prescription, over a six-week clinical trial. The results indicated that the 4% BP cream was effective and well-tolerated. Only 11.6% of participants reported moderate-to-severe discomfort, and clinicians identified side effects in 53.8% of all patients. Specifically, 53.8% of patients reported no irritation, 41.4% reported some irritation, and only 4.8% experienced bothersome irritation. Most patients showed a very satisfactory response after six weeks and expressed eagerness to continue the treatment. A satisfactory response was observed as early as two weeks, supporting the hypothesis that the hydrophase formulation of Brevoxyl helps improve efficacy and reduce irritation associated with benzoyl peroxide use [173].

In another study, Weinberg et al. indicated that benzoyl peroxide formulated in hydrophase bases (Brevoxyl Creamy Washes and Gels) exhibits significant efficacy in acne treatment with less irritancy compared to other benzoyl peroxide preparations. It is believed that this product's reduced irritancy stems from its unique delivery vehicle, which includes dimethyl isosorbide that dissolves benzoyl peroxide crystals upon application to the skin [173].

## 5. Future Perspective

Based on the gathered literature, various strategies have been identified to effectively reduce the adverse effects associated with retinol application. These strategies offer alternative drug release mechanisms, including controlled and sustained release, enabling gradual and regulated drug molecule release over an extended period. This controlled release mechanism helps minimize the likelihood of irritation from active substances with known irritant properties, such as retinoids. Additionally, these approaches can enhance the stability of retinoids within formulations and promote improved skin penetration, thereby reducing irritating effects.

Given the beneficial outcomes of advanced retinoid formulations in mitigating retinoid-induced irritation, incorporating these approaches into formulation design appears promising, considering their proven advantages in both in vivo and in vitro settings. Further research is needed to develop novel interventions aimed at reducing skin irritation caused by retinoids, thus expanding the range of available application methods. In this context, we present several methodologies designed to mitigate retinoid-induced irritation, which warrant further investigation and exploration. Numerous studies have demonstrated the efficacy of these interventions in reducing skin irritation resulting from topical and transdermal application of active compounds.

Further investigation is necessary to advance novel interventions aimed at reducing the adverse effects of retinoid-induced skin irritation, thereby expanding the available range of application methods. Based on the preceding discussion, it can be inferred that multiple drug delivery strategies have the potential to mitigate the adverse effects associated with retinoid use. Currently, the predominant technology focus is on transdermal administration rather than topical application. This observation may warrant consideration in future investigations related to the irritative consequences associated with retinol use.

## 6. Conclusion

Various techniques have been employed in formulation to enhance drug delivery systems, including encapsulation of retinoids, conversion of retinoids into nanoparticles, formation of complexes with cyclodextrin, and binding of retinoids with carriers, such as polymers, NLC, and SLN. Ongoing research is exploring the incorporation of elements that provide anti-irritation properties, strengthen the skin barrier, and enhance skin hydration into retinoid formulations. These components include glucosamine, trehalose, ectoine, sucralfate, omega-9, and 4-t-butylcyclohexanol. Additionally, an ethanolic bark extract derived from *Alstonia scholaris* R. Br. is also included. The aforementioned methodologies highlight that nonlamellar liquid crystalline (NLC) and solid lipid nanoparticle (SLN) drug delivery technologies are widely employed to mitigate the irritative effects associated with retinoid administration. One could argue that the use of nanoparticle lipid carriers (NLC) offers more advantages compared to solid lipid nanoparticles (SLN) because NLC represents an enhanced version of SLN. The development of NLC was prompted by the need to address significant deficiencies identified within SLN formulations.

## Figures and Tables

**Table 1:** Mechanism and skin irritation test method of strategy to reduce retinoid-induced skin irritation.

Strategy	Retinoids	Mechanism	Skin irritation test	References
Encapsulated nanoparticle	Retinol propionate and hydroxypinacolone retinoate	Encapsulation using a high-pressure homogenization technique	In vivo test on rabbits	[8]
Encapsulation into solid lipid nanoparticles (SLNs)	Adapalene	Encapsulation in solid lipid nanoparticles (SLNs) using an ion-pairing strategy	In vivo testing on female rats	[34]
Encapsulation and controlled release silicone particle	Retinol	Encapsulation of retinol with silicone particles	In vivo test on humans	[35]
NLC (nanostructured lipid carriers)	Tretinoin	NLC (nanostructured lipid carriers) loaded with tretinoin were developed using stearic acid, Span 60, oleic acid, and Tween 80 incorporated into carbopol gel	In vivo test on rats	[36]
Solid lipid nanoparticles (SLN)	Vitamin A	Dispersion of solid lipid nanoparticles using a high-pressure homogenization technique	In vivo test on rabbits	[37]
Nanostructured lipid carriers (NLC)	Tretinoin	Liposomes loaded with TRE were developed using nanostructured lipid carriers (NLC) as the lipid carrier	In vivo testing on female lacquer mice	[38]
Liposome	Tretinoin	Encapsulation of tretinoin into liposomes using a modified ether injection method	In vivo test on humans	[39]
Ion-pairing strategy in solid lipid nanoparticle	Tretinoin	Encapsulation of tretinoin into liposomes using a modified ether injection method	In vivo testing on female rats	[40]
Nanostructured lipid carriers	Retinyl palmitate	Lipid nanocarrier formulated with retinyl palmitate	In vitro test on RHE model (human epidermis)	[41]
Nanostructured lipid carriers	Adapalene	ADA-loaded nanostructured lipid carriers (NLC-ADA) were formed using a probe sonicator	In vivo test on humans	[42]
Niosome	Retinaldehyde	Nanoemulsion retinaldehyde-loaded niosome	In vivo test on humans	[43]
Proniosome	Tretinoin	Niosomes can be created right before usage by hydrating proniosomes	In vivo test on humans	[44]
Nanoemulsion	Adapalene	The combination that developed using nanoemulsion technology. Combination of adapalene and clindamycin w/w	In vivo test on humans	[45]
Conjugated polymer	All-trans-retinoic acid (ATRA)	Formation of polymer conjugate by covalently binding all-trans-retinoic acid through ester linkage with polyvinyl alcohol (PVA)	In vivo on rats	[46]

*Addition of ingredients*				
Glucosamine, trehalose, ectoine, sucralfate, omega-9, and 4-t-butylcyclohexanol	Retinol	Formulation of retinol with ingredients that can genetically inhibit irritation	In vivo test on humans	[4]
Polyolprepolymer-2	Tretinoin	Combination of tretinoin with polyolprepolymer-2	In vivo testing on guinea pigs	[47]
	Tretinoin	Combination of tretinoin with polyolprepolymer-2	In vivo test on humans	[48]
Addition of cyclodextrin	Retinoic acid	Complexation forming cyclodextrin beta complex (*β*-CD)	In vivo test on humans	[49]
	Isotretinoin	Isotretinoin HP-*β*-CD complex is made using the freeze-drying method	In vivo test on rabbits	[50]
*Alstonia scholaris* extract	All-trans-retinoic acid (ATRA)	Addition of 0.1% *Alstonia scholaris* extract to 2500 IU retinol cream	In vivo test on humans	[51]

## Data Availability

All relatable data can be requested upon reasonable request from the corresponding author.

## References

[1] Hubbard B. A., Unger J. G., Rohrich R. J. (2014). Reversal of skin aging with topical retinoids. *Plastic and Reconstructive Surgery*.

[2] Motamedi M., Chehade A., Sanghera R., Grewal P. (2022). A clinician’s guide to topical retinoids. *Journal of Cutaneous Medicine and Surgery*.

[3] Milosheska D., Roškar R. (2022). Use of retinoids in topical antiaging treatments: a focused review of clinical evidence for conventional and nanoformulations. *Advances in Therapy*.

[4] Kang S., Kim K., Jun S. H. (2021). Anti-irritant strategy against retinol based on the genetic analysis of Korean population: a genetically guided top-down approach. *Pharmaceutics*.

[5] Wanichwecharungruang S., Pisetpackdeekul P., Supmuang P. (2016). Proretinal nanoparticles: stability, release, efficacy, and irritation. *International Journal of Nanomedicine*.

[6] Frieling G. W., Tegeder A. R. (2015). Cutaneous reactions to retinoids. *Cutaneous Drug Eruptions*.

[7] Mukherjee S., Date A., Patravale V., Korting H. C., Roeder A., Weindl G. (2006). Retinoids in the treatment of skin aging: an overview of clinical efficacy and safety. *Clinical Interventions in Aging*.

[8] Bai D., Hu F., Xu H., Huang J., Wu C., Zhang J. (2023). High stability and low irritation of retinol propionate and hydroxypinacolone retinoate supramolecular nanoparticles with effective anti-wrinkle efficacy. *Pharmaceutics*.

[9] Kim B. H., Lee Y. S., Kang K. S. (2003). The mechanism of retinol-induced irritation and its application to anti-irritant development. *Toxicology Letters*.

[10] Czajkowska-Kośnik A., Szekalska M., Winnicka K. (2019). Nanostructured lipid carriers: a potential use for skin drug delivery systems. *Pharmacological Reports*.

[11] Zasada M., Budzisz E. (2019). Retinoids: active molecules influencing skin structure formation in cosmetic and dermatological treatments. *Advances in Dermatology and Allergology*.

[12] Kim B. H. (2010). Safety evaluation and anti-wrinkle effects of retinoids on skin. *Toxicological Research*.

[13] Poon F., Kang S., Chien A. L. (2015). Mechanisms and treatments of photoaging. *Photodermatology, Photoimmunology and Photomedicine*.

[14] Rossetti D., Kielmanowicz M. G., Vigodman S. (2011). A novel anti-ageing mechanism for retinol: induction of dermal elastin synthesis and elastin fibre formation. *International Journal of Cosmetic Science*.

[15] Geiger J. M., Hommel L., Harms M., Saurat J. H. (1996). Oral 13-cis retinoic acid is superior to 9-cis retinoic acid in sebosuppression in human beings. *Journal of the American Academy of Dermatology*.

[16] Sarkar R., Arora P., Garg K. V. (2013). Cosmeceuticals for hyperpigmentation: what is available?. *Journal of Cutaneous and Aesthetic Surgery*.

[17] Gollnick H. P. M. (2015). From new findings in acne pathogenesis to new approaches in treatment. *Journal of the European Academy of Dermatology and Venereology*.

[18] Czernielewski J., Michel S., Bouclier M., Baker M., Hensby C. (2001). Adapalene biochemistry and the evolution of a new topical retinoid for treatment of acne. *Journal of the European Academy of Dermatology and Venereology*.

[19] Corbeil J., Rapaport E., Richman D. D., Looneyt D. J., Veterans I. D. (1994). Antiproliferative effect of retinoid compounds on kaposi’s sarcoma cells. *ournal of Clinical Investigation*.

[20] Hoover L. L., Burton E. G., O’Neill M. L. (2008). Retinoids regulate TGF*β* signaling at the level of Smad2 phosphorylation and nuclear accumulation. *Biochimica et Biophysica Acta (BBA) - Molecular Cell Research*.

[21] Kolli S. S., Pecone D., Pona A., Cline A., Feldman S. R. (2019). Topical retinoids in acne vulgaris: a systematic review. *American Journal of Clinical Dermatology*.

[22] Huang S. S., Huang J. S. (2005). TGF-*β* control of cell proliferation. *Journal of Cellular Biochemistry*.

[23] Antman K., Chang Y. (2000). Kaposi’s sarcoma. *New England Journal of Medicine*.

[24] Shao Y., He T., Fisher G. J., Voorhees J. J., Quan T. (2017). Molecular basis of retinol anti-ageing properties in naturally aged human skin in vivo. *International Journal of Cosmetic Science*.

[25] Chandraratna R. A. S. (1997). Tazarotene: the first receptor-selective topical retinoid for the treatment of psoriasis. *Journal of the American Academy of Dermatology*.

[26] Lowe M. N., Plosker G. L. (2000). Bexarotene. *American Journal of Clinical Dermatology*.

[27] Wolverton S., Wu J., Wu J. (2021). *Comprehensive Dermatologic Drug Therapy*.

[28] Szymański Ł, Skopek R., Palusińska M. (2020). Retinoic acid and its derivatives in skin. *Cells*.

[29] Bolognia J. L., Jorizzo J. L., Schaffer J. V. (2012). *Dermatology*.

[30] Gollnick H., Schramm M. (1998). Topical therapy in acne. *Journal of the European Academy of Dermatology and Venereology: JEADV*.

[31] Kahraman E., Kaykın M., Şahin Bektay H., Güngör S. (2019). Recent advances on topical application of ceramides to restore barrier function of skin. *Cosmetics*.

[32] Zhang Z., Tsai P. C., Ramezanli T., Michniak-Kohn B. B. (2013). Polymeric nanoparticles-based topical delivery systems for the treatment of dermatological diseases. *WIREs Nanomedicine and Nanobiotechnology*.

[33] Gonçalves A., Estevinho B. N., Rocha F. (2019). Formulation approaches for improved retinoids delivery in the treatment of several pathologies. *European Journal of Pharmaceutics and Biopharmaceutics*.

[34] Rodrigues L. B. O., Lima F. A., Alves C. P. B. (2020). Ion pair strategy in solid lipid nanoparticles: a targeted approach to improve epidermal targeting with controlled adapalene release, resulting reduced skin irritation. *Pharmaceutical Research*.

[35] Shields C. W., White J. P., Osta E. G. (2018). Encapsulation and controlled release of retinol from silicone particles for topical delivery. *Journal of Controlled Release*.

[36] Ghate V. M., Lewis S. A., Prabhu P., Dubey A., Patel N. (2016). Nanostructured lipid carriers for the topical delivery of tretinoin. *European Journal of Pharmaceutics and Biopharmaceutics*.

[37] Pople P. V., Singh K. K. (2006). Development and evaluation of topical formulation containing solid lipid nanoparticles of vitamin A. *AAPS PharmSciTech*.

[38] Raza K., Singh B., Lohan S. (2013). Nano-lipoidal carriers of tretinoin with enhanced percutaneous absorption, photostability, biocompatibility and anti-psoriatic activity. *International Journal of Pharmaceutics*.

[39] Rahman S. A., Abdelmalak N. S., Badawi A., Elbayoumy T., Sabry N., El Ramly A. (2015). Tretinoin-loaded liposomal formulations: from lab to comparative clinical study in acne patients. *Drug Delivery*.

[40] Castro G. A., Coelho A. L. L. R., Oliveira C. A., Mahecha G. A. B., Oréfice R. L., Ferreira L. A. M. (2009). Formation of ion pairing as an alternative to improve encapsulation and stability and to reduce skin irritation of retinoic acid loaded in solid lipid nanoparticles. *International Journal of Pharmaceutics*.

[41] Pinto F., Fonseca L. P., Souza S., Oliva A., de Barros D. P. C. (2020). Topical distribution and efficiency of nanostructured lipid carriers on a 3D reconstructed human epidermis model. *Journal of Drug Delivery Science and Technology*.

[42] Ahmad Nasrollahi S., Koohestani F., Naeimifar A., Samadi A., Vatanara A., Firooz A. (2021). Preparation and evaluation of adapalene nanostructured lipid carriers for targeted drug delivery in acne. *Dermatologic Therapy*.

[43] Kim J., Kim J., Lee Y. I., Suk J., Lee D., Lee J. H. (2021). A pilot study evaluating the efficacy and safety of retinaldehyde-loaded niosomes against mild-to-moderate acne. *Journal of Cosmetic Dermatology*.

[44] Rahman S. A., Abdelmalak N. S., Badawi A., Elbayoumy T., Sabry N., Ramly A. E. (2015). Formulation of tretinoin-loaded topical proniosomes for treatment of acne: in-vitro characterization, skin irritation test and comparative clinical study. *Drug Delivery*.

[45] Kubavat A., Modi A., Bajaj B. (2012). Efficacy and safety of a nano-emulsion gel formulation of adapalene 0.1% and clindamycin 1% combination in acne vulgaris: a randomized, open label, active-controlled, multicentric, phase IV clinical trial. *Indian Journal of Dermatology Venereology and Leprology*.

[46] Castleberry S. A., Quadir M. A., Sharkh M. A., Shopsowitz K. E., Hammond P. T. (2017). Polymer conjugated retinoids for controlled transdermal delivery. *Journal of Controlled Release*.

[47] Quigley J. W., Bucks D. A. W. (1998). Reduced skin irritation with tretinoin containing polyolprepolymer-2, a new topical tretinoin delivery system: a summary of preclinical and clinical investigations. *Journal of the American Academy of Dermatology*.

[48] Luckya A. W., Cullenb S. I., Jarratt M. T., Quigley J. W. (1998). Comparative efficacy and safety of two 0.025% tretinoin gels: results from a multicenter double-blind, parallel study. *Journal of the American Academy of Dermatology*.

[49] Anadolu R. Y., Sen T., Tarimci N., Birol A., Erdem C. (2004). Improved efficacy and tolerability of retinoic acid in acne vulgaris: a new topical formulation with cyclodextrin complex *ψ*. *Journal of the European Academy of Dermatology and Venereology*.

[50] Kaur N., Puri R., Jain S. K. (2010). Drug-cyclodextrin-vesicles dual carrier approach for skin targeting of anti-acne agent. *AAPS PharmSciTech*.

[51] Lee S. J., Cho S. A., An S. S. (2012). *Alstonia scholaris* R. Br. significantly inhibits retinoid-induced skin irritation *in vitro* and *in vivo*. *Evidence-Based Complementary and Alternative Medicine*.

[52] Drachuk I., Gupta M. K., Tsukruk V. V. (2013). Biomimetic coatings to control cellular function through cell surface engineering. *Advanced Functional Materials*.

[53] Mandal B., Bhattacharjee H., Mittal N. (2013). Core–shell-type lipid–polymer hybrid nanoparticles as a drug delivery platform. *Nanomedicine: Nanotechnology, Biology and Medicine*.

[54] Sivadasan D., Sultan M. H., Madkhali O., Almoshari Y., Thangavel N. (2021). Polymeric lipid hybrid nanoparticles (PLNs) as emerging drug delivery platform—a comprehensive review of their properties, preparation methods, and therapeutic applications. *Pharmaceutics*.

[55] Gulati M., Grover M., Singh S., Singh M. (1998). Lipophilic drug derivatives in liposomes. *International Journal of Pharmaceutics*.

[56] (2018). Liposome drug products chemistry, manufacturing, and controls; human pharmacokinetics and bioavailability; and labeling documentation guidance for industry. https://www.fda.gov/Drugs/GuidanceComplianceRegulatoryInformation/Guidances/default.htm.

[57] Ibaraki H., Kanazawa T., Oogi C., Takashima Y., Seta Y. (2019). Effects of surface charge and flexibility of liposomes on dermal drug delivery. *Journal of Drug Delivery Science and Technology*.

[58] Liang T., Zhang R., Liu X. (2021). Recent advances in macrophage-mediated drug delivery systems. *International Journal of Nanomedicine*.

[59] Liu P., Chen G., Zhang J. (2022). A review of liposomes as a drug delivery system: current status of approved products, regulatory environments, and future perspectives. *Molecules*.

[60] Shaji J., Iyer S. (2012). Preparation, optimization and in-vivo hepatoprotective evaluation of quercetin liposomes. *International Journal of Current Pharmaceutical Research*.

[61] Arnardóttir H. B., Sveinsson S. J., Kristmundsdóttir T. (1996). The release of clindamycin phosphate from a suspension of different types of liposomes and selected topical dosages forms. *International Journal of Pharmaceutics*.

[62] Daraee H., Etemadi A., Kouhi M., Alimirzalu S., Akbarzadeh A. (2014). Application of liposomes in medicine and drug delivery. *Artificial Cells, Nanomedicine, and Biotechnology*.

[63] Fielding R. M. (1991). Liposomal drug delivery. Advantages and limitations from a clinical pharmacokinetic and therapeutic perspective. *Clinical Pharmacokinetics*.

[64] Ge X., Wei M., He S., Yuan W. E. (2019). Advances of non-ionic surfactant vesicles (niosomes) and their application in drug delivery. *Pharmaceutics*.

[65] Chen S., Hanning S., Falconer J., Locke M., Wen J. (2019). Recent advances in non-ionic surfactant vesicles (niosomes): Fabrication, characterization, pharmaceutical and cosmetic applications. *European Journal of Pharmaceutics and Biopharmaceutics*.

[66] Cosco D., Paolino D., Muzzalupo R. (2009). Novel PEG-coated niosomes based on bola-surfactant as drug carriers for 5-fluorouracil. *Biomedical Microdevices*.

[67] Nasr M., Mansour S., Mortada N. D., Elshamy A. A. (2008). Vesicular aceclofenac systems: a comparative study between liposomes and niosomes. *Journal of Microencapsulation*.

[68] Jain C. P., Vyas S. P., Dixit V. K. (2006). Niosomal system for delivery of rifampicin to lymphatics. *Indian Journal of Pharmaceutical Sciences*.

[69] Naresh R. A. R., Pillai G. K., Udupa N., Chandrashekar G. (1994). Anti-inflammatory activity of niosome encapsulated diclofenac sodium in arthritic rats. *Indian Journal of Pharmacology*.

[70] Bayindir Z. S., Yuksel N. (2010). Characterization of niosomes prepared with various nonionic surfactants for paclitaxel oral delivery. *Journal of Pharmaceutical Sciences*.

[71] Arafa M. G., Ayoub B. M. (2017). DOE optimization of nano-based carrier of pregabalin as hydrogel: new therapeutic and chemometric approaches for controlled drug delivery systems. *Scientific Reports*.

[72] Namdeo A., Jain N. K. (2023). Niosomes as drug carriers. *Indian Journal of Pharmaceutical Sciences*.

[73] Uchegbu I. F., Vyas S. P. (1998). Non-ionic surfactant based vesicles (niosomes) in drug delivery. *International Journal of Pharmaceutics*.

[74] Rahimpour Y., Hamishehkar H. (2012). Liposomes in cosmeceutics. *Expert Opinion on Drug Delivery*.

[75] Rahimpour Y., Hamishehkar H. (2012). Niosomes as carrier in dermal drug delivery. *Recent Advances in Novel Drug Carrier Systems*.

[76] Verma A. K., Bindal M. (2012). A review on niosomes: an ultimate controlled and novel drug delivery carrier. *International Journal of Nanoparticles*.

[77] Hofland H. E. J., van der Geest R., Bodde H. E., Junginger H. E., Bouwstra J. A. (1994). Estradiol permeation from nonionic surfactant vesicles through human stratum corneum in vitro. *Pharmaceutical Research*.

[78] Paliwal R., Paliwal S. R., Kenwat R., Kurmi B. D., Sahu M. K. (2020). Solid lipid nanoparticles: a review on recent perspectives and patents. *Expert Opinion on Therapeutic Patents*.

[79] Khosa A., Reddi S., Saha R. N. (2018). Nanostructured lipid carriers for site-specific drug delivery. *Biomedicine and Pharmacotherapy*.

[80] Battaglia L., Serpe L., Muntoni E., Zara G. P., Trotta M., Gallarate M. (2011). Methotrexate-loaded SLNs prepared by coacervation technique: in vitro cytotoxicity and in vivo pharmacokinetics and biodistribution. *Nanomedicine*.

[81] Lai F., Sinico C., De Logu A., Zaru M., Müller R. H., Fadda A. M. (2007). SLN as a topical delivery system for Artemisia arborescens essential oil: in vitro antiviral activity and skin permeation study. *International Journal of Nanomedicine*.

[82] Souto E. B., Baldim I., Oliveira W. P. (2020). SLN and NLC for topical, dermal, and transdermal drug delivery. *Expert Opinion on Drug Delivery*.

[83] Martel-Estrada S. A., Morales-Cardona A. I., Vargas-Requena C. L., Rubio-Lara J. A., Martínez-Pérez C. A., Jimenez-Vega F. (2022). Delivery systems in nanocosmeceuticals. *Reviews on Advanced Materials Science*.

[84] Muchtaridi M., Hidayat S., Ibrahim F., Suhandi C. (2022). A systematic review: molecular docking simulation of small molecules as anticancer non-small cell lung carcinoma drug candidates. *Journal of Advanced Pharmaceutical Technology & Research*.

[85] Suhandi C., Alfathonah S. S., Hasanah A. N. (2023). Potency of xanthone derivatives from *Garcinia mangostana* L. for COVID-19 treatment through angiotensin-converting enzyme 2 and main protease blockade: a computational study. *Molecules*.

[86] Suhandi C., Wilar G., Lesmana R. (2023). Propolis-based nanostructured lipid carriers for *α*-mangostin delivery: formulation, characterization, and in vitro antioxidant activity evaluation. *Molecules*.

[87] Beloqui A., Solinís M. Á, Rodríguez-Gascón A., Almeida A. J., Préat V. (2016). Nanostructured lipid carriers: promising drug delivery systems for future clinics. *Nanomedicine: Nanotechnology, Biology and Medicine*.

[88] Shidhaye S., Vaidya R., Sutar S., Patwardhan A., Kadam V. (2008). Solid lipid nanoparticles and nanostructured lipid carriers--innovative generations of solid lipid carriers. *Current Drug Delivery*.

[89] Müller R. H., Shegokar R., Keck C. M. (2011). 20 years of lipid nanoparticles (SLN and NLC): present state of development and industrial applications. *Current Drug Discovery Technologies*.

[90] Battaglia L., Gallarate M. (2012). Lipid nanoparticles: state of the art, new preparation methods and challenges in drug delivery. *Expert Opinion on Drug Delivery*.

[91] Gordillo-Galeano A., Mora-Huertas C. E. (2018). Solid lipid nanoparticles and nanostructured lipid carriers: a review emphasizing on particle structure and drug release. *European Journal of Pharmaceutics and Biopharmaceutics*.

[92] Waghule T., Rapalli V. K., Gorantla S. (2020). Nanostructured lipid carriers as potential drug delivery systems for skin disorders. *Current Pharmaceutical Design*.

[93] Muchow M., Maincent P., Müller R. H. (2008). Lipid nanoparticles with a solid matrix (SLN®, NLC®, LDC®) for oral drug delivery. *Drug Development and Industrial Pharmacy*.

[94] Seyfoddin A., Shaw J., Al-Kassas R. (2010). Solid lipid nanoparticles for ocular drug delivery. *Drug Delivery*.

[95] Sung Y. K., Kim S. W. (2020). Recent advances in polymeric drug delivery systems. *Biomaterials Research*.

[96] Lombardo D., Kiselev M. A., Caccamo M. T. (2019). Smart nanoparticles for drug delivery application: development of versatile nanocarrier platforms in biotechnology and nanomedicine. *Journal of Nanomaterials*.

[97] Srivastava A., Yadav T., Sharma S., Nayak A., Akanksha Kumari A., Mishra N. (2016). Polymers in drug delivery. *Journal of Biosciences and Medicines*.

[98] Kleinstreuer C., Childress E., Kennedy A. (2013). Targeted drug delivery: multifunctional nanoparticles and direct micro-drug delivery to tumors. *Transport in Biological Media*.

[99] Yadav K., Singh M. R., Rai V. K., Srivastava N., Prasad Yadav N. (2020). Commercial aspects and market potential of novel delivery systems for bioactives and biological agents. *Advances and Avenues in the Development of Novel Carriers for Bioactives and Biological Agents*.

[100] Ulbrich K., Holá K., Šubr V., Bakandritsos A., Tuček J., Zbořil R. (2016). Targeted drug delivery with polymers and magnetic nanoparticles: covalent and noncovalent approaches, release control, and clinical studies. *Chemistry Review*.

[101] Liechty W. B., Kryscio D. R., Slaughter B. V., Peppas N. A. (2010). Polymers for drug delivery systems. *Annual Review of Chemical and Biomolecular Engineering*.

[102] Priya V. S. V., Roy H. K., jyothi N., Prasanthi N. L. (2016). Polymers in drug delivery technology, types of polymers and applications. *Scholars Academic Journal of Pharmacy*.

[103] Mitchell M. J., Billingsley M. M., Haley R. M., Wechsler M. E., Peppas N. A., Langer R. (2020). Engineering precision nanoparticles for drug delivery. *Nature Reviews Drug Discovery*.

[104] Anagnostou K., Stylianakis M., Michaleas S., Skouras A. (2020). Biodegradable nanomaterials. *Nanomaterials for Clinical Applications: Case Studies in Nanomedicines*.

[105] Singh N., Joshi A., Toor A. P., Verma G. (2017). Drug delivery: advancements and challenges. *Nanostructures for Drug Delivery*.

[106] Skov M. J., Quigley J. W., Bucks D. A. W. (1997). Topical delivery system for tretinoin: research and clinical implications. *Journal of Pharmaceutical Sciences*.

[107] Niemiec S. M., Wu H. L., Jayaraman S. (1997). Effect of polyolprepolymer on the deposition of retinoic acid in various strata of hamster ear following topicalin vivo application of gel formulations: Correlation with disposition in human skin. *Drug Delivery*.

[108] Lipinski C. A., Discovery M. (2002). Poor aqueous solubility-An industry wide problem in drug discovery. https://www.researchgate.net/publication/279892668.

[109] Larson N., Ghandehari H. (2012). Polymeric conjugates for drug delivery. *Chemistry of Materials*.

[110] Pasut G., Veronese F. M. (2007). Polymer–drug conjugation, recent achievements and general strategies. *Progress in Polymer Science*.

[111] R Pomalingo D., Suhandi C., Megantara S., Muchtaridi M. (2021). The optimization of *α*-mangostin as a new drug candidate through molecular docking and dynamic simulations. *Rasayan Journal of Chemistry*.

[112] Suharyani I., Suhandi C., Rizkiyan Y. (2023). Molecular docking in prediction of *α*-mangostin/cyclodextrin inclusion complex formation. *AIP Conference Proceedings*.

[113] Miranda J. C. d, Martins T. E. A., Veiga F., Ferraz H. G. (2011). Cyclodextrins and ternary complexes: technology to improve solubility of poorly soluble drugs. *Brazilian Journal of Pharmaceutical Sciences*.

[114] Braga S. S., Pais J. (2018). Getting under the skin: cyclodextrin inclusion for the controlled delivery of active substances to the dermis. *Design of Nanostructures for Versatile Therapeutic Applications*.

[115] Dhiman P., Bhatia M. (2020). Pharmaceutical applications of cyclodextrins and their derivatives. *Journal of Inclusion Phenomena and Macrocyclic Chemistry*.

[116] Rasheed A., Kumar C. K. A., Vvnss S. (2008). Cyclodextrins as drug carrier molecule: a review. *Scientia Pharmaceutica*.

[117] Loftsson T., Brewster M. E. (2011). Pharmaceutical applications of cyclodextrins: effects on drug permeation through biological membranes. *Journal of Pharmacy and Pharmacology*.

[118] Furuishi T., Takahashi S., Ogawa N. (2017). Enhanced dissolution and skin permeation profiles of epalrestat with *β*-cyclodextrin derivatives using a cogrinding method. *European Journal of Pharmaceutical Sciences*.

[119] Ferreira L., Mascarenhas-Melo F., Rabaça S. (2023). Cyclodextrin-based dermatological formulations: dermopharmaceutical and cosmetic applications. *Colloids and Surfaces B: Biointerfaces*.

[120] Loftsson T., Duchêne D. (2007). Cyclodextrins and their pharmaceutical applications. *International Journal of Pharmaceutics*.

[121] Loftsson T., Masson M. (2001). Cyclodextrins in topical drug formulations: theory and practice. *International Journal of Pharmaceutics*.

[122] Cal K., Centkowska K. (2008). Use of cyclodextrins in topical formulations: practical aspects. *European Journal of Pharmaceutics and Biopharmaceutics*.

[123] Pouton C. W. (1997). Formulation of self-emulsifying drug delivery systems. *Advanced Drug Delivery Reviews*.

[124] Wooster T. J., Labbett D., Sanguansri P., Andrews H. (2016). Impact of microemulsion inspired approaches on the formation and destabilisation mechanisms of triglyceride nanoemulsions. *Soft Matter*.

[125] Mason T. G., Wilking J. N., Meleson K., Chang C. B., Graves S. M. (2006). Nanoemulsions: formation, structure, and physical properties. *Journal of Physics: Condensed Matter*.

[126] Kale S. N., Deore S. L. (2016). Emulsion micro emulsion and nano emulsion: a review. *Systematic Reviews in Pharmacy*.

[127] Samson S., Basri M., Fard Masoumi H. R., Abedi Karjiban R., Abdul Malek E. (2016). Design and development of a nanoemulsion system containing copper peptide by D-optimal mixture design and evaluation of its physicochemical properties. *RSC Advances*.

[128] Che Marzuki N. H., Wahab R. A., Abdul Hamid M. (2019). An overview of nanoemulsion: concepts of development and cosmeceutical applications. *Biotechnology and Biotechnological Equipment*.

[129] Anton N., Vandamme T. F. (2011). Nano-emulsions and micro-emulsions: clarifications of the critical differences. *Pharmaceutical Research*.

[130] Praveen Kumar G., Divya A., Kumar G. P. (2015). Nanoemulsion based targeting in cancer therapeutics. *Medicinal Chemistry*.

[131] Amiji M., Tiwari S. (2006). Nanoemulsion formulations for tumor-targeted delivery. *Nanotechnology for Cancer Therapy*.

[132] Kaci M., Belhaffef A., Meziane S. (2018). Nanoemulsions and topical creams for the safe and effective delivery of lipophilic antioxidant coenzyme Q10. *Colloids and Surfaces B: Biointerfaces*.

[133] Afornali A., Lorencini M. (2016). Nanoemulsions to prevent photoaging. *Nanoscience in Dermatology*.

[134] Lieberman H., Rieger M., Banker G. S. (2010). *Pharmaceutical Dosage Forms: Disperse Systems*.

[135] Rübsam M., Mertz A. F., Kubo A. (2017). E-cadherin integrates mechanotransduction and EGFR signaling to control junctional tissue polarization and tight junction positioning. *Nature Communications*.

[136] Tran Q. T., Kennedy L. H., Leon Carrion S. (2012). EGFR regulation of epidermal barrier function. *Physiological Genomics*.

[137] Eleutherio E., Araújo Brasil A., Breves Rona G., Stfhany Silva Magalhães R. (2018). Trehalose: as sweet as effective in biomedical research and biotechnology. *Advances in Biotechnology & Microbiology*.

[138] Bissett D. L. (2006). Glucosamine: an ingredient with skin and other benefits. *Journal of Cosmetic Dermatology*.

[139] Galinski E. A., Pfeiffer H., Trüper H. G. (1985). 1,4,5,6-Tetrahydro-2-methyl-4-pyrimidinecarboxylic acid. A novel cyclic amino acid from halophilic phototrophic bacteria of the genus Ectothiorhodospira. *European Journal of Biochemistry*.

[140] Lentzen G., Schwarz T. (2006). Extremolytes: natural compounds from extremophiles for versatile applications. *Applied Microbiology and Biotechnology*.

[141] Kauth M., Trusova O. V. (2022). Topical ectoine application in children and adults to treat inflammatory diseases associated with an impaired skin barrier: a systematic review. *Dermatologic Therapy*.

[142] Rieckmann T., Gatzemeier F., Christiansen S., Rothkamm K., Münscher A. (2019). The inflammation-reducing compatible solute ectoine does not impair the cytotoxic effect of ionizing radiation on head and neck cancer cells. *Scientific Reports*.

[143] Yu I., Nagaoka M. (2004). Slowdown of water diffusion around protein in aqueous solution with ectoine. *Chemical Physics Letters*.

[144] Suhandi C., Mohammed A. F. A., Wilar G., El-Rayyes A., Wathoni N. (2023). Effectiveness of mesenchymal stem cell secretome on wound healing: a systematic review and meta-analysis. *Tissue Engineering and Regenerative Medicine*.

[145] Megantara S., Wathoni N., Mohammed A. F. A., Suhandi C., Ishmatullah M. H., Putri M. F. F. D. (2022). In silico study: combination of *α*-mangostin and chitosan conjugated with trastuzumab against human epidermal growth factor receptor 2. *Polymers*.

[146] TingTing H., Yun S., LiPing Z., Wen G., GaoXiong R. (2009). Flavonoids in leaves of Alstonia scholaris. *China Journal of Chinese Materia Medica*.

[147] Wang F., Ren F. C., Liu J. K. (2009). Alstonic acids A and B, unusual 2,3-secofernane triterpenoids from Alstonia scholaris. *Phytochemistry*.

[148] Lee S. J., Shin E. J., Son K. H., Chang H. W., Kang S. S., Kim H. P. (1995). Anti-inflammatory activity of the major constituents of Lonicera japonica. *Archives of Pharmacal Research*.

[149] Duplan H., Nocera T. (2018). [Skin hydration and hydrating products]. *Annales de Dermatologie et de Vénéréologie*.

[150] Dąbrowska A. K., Spano F., Derler S., Adlhart C., Spencer N. D., Rossi R. M. (2018). The relationship between skin function, barrier properties, and body-dependent factors. *Skin Research and Technology*.

[151] De Oliveira A. C., Morocho-Jácome A. L., De Castro Lima C. R. (2021). Cosmetics applications. *Microalgae: Cultivation, Recovery of Compounds and Applications*.

[152] Ratz-Lyko A., Arct J., Pytkowska K. (2016). Moisturizing and antiinflammatory properties of cosmetic formulations containing Centella asiatica extract. *Indian Journal of Pharmaceutical Sciences*.

[153] Camargo Junior F. B. d, Gaspar L. R., Campos P. M. B. G. M. (2012). Immediate and long-term effects of polysaccharides-based formulations on human skin. *Brazilian Journal of Pharmaceutical Sciences*.

[154] Dal’Belo S. E., Rigo Gaspar L., Berardo Gonçalves Maia Campos P. M. (2006). Moisturizing effect of cosmetic formulations containing Aloe vera extract in different concentrations assessed by skin bioengineering techniques. *Skin Research and Technology*.

[155] Shankar R., Upadhyay P. K., Kumar M. (2021). Invasomes for enhanced delivery through the skin: evaluation of systems to meet with clinical challenges. *Pharmaceutical Nanotechnology*.

[156] Babaie S., Bakhshayesh A. R. D., Ha J. W., Hamishehkar H., Kim K. H. (2020). Invasome: a novel nanocarrier for transdermal drug delivery. *Nanomaterials*.

[157] Vanić Ž (2014). Phospholipid vesicles for enhanced drug delivery in dermatology. *Journal of Drug Discovery, Development and Delivery*.

[158] Jain S., Tripathi S., Tripathi P. K. (2022). Antiarthritic potential of berberine loaded invasomal gel. *Phytomedicine Plus*.

[159] Bajaj A., Malhotra G., Madan M., Amrutiya N., Bakshi A. (2008). A novel metered dose transdermal spray formulation for oxybutynin. *Indian Journal of Pharmaceutical Sciences*.

[160] Zhuang C., Zhong Y., Zhao Y. (2019). Effect of deacetylation degree on properties of Chitosan films using electrostatic spraying technique. *Food Control*.

[161] Ranade S., Bajaj A., Londhe V., Babul N., Kao D. (2017). Fabrication of topical metered dose film forming sprays for pain management. *European Journal of Pharmaceutical Sciences*.

[162] Umar A. K., Butarbutar M., Sriwidodo S., Wathoni N. (2020). Film-forming sprays for topical drug delivery. *Drug Design, Development and Therapy*.

[163] McAuley W. J., Caserta F. (2015). Film-forming and heated systems. *Novel Delivery Systems for Transdermal and Intradermal Drug Delivery*.

[164] Frederiksen K., Guy R. H., Petersson K. (2015). The potential of polymeric film-forming systems as sustained delivery platforms for topical drugs. *Expert Opinion on Drug Delivery*.

[165] Lu W., Luo H., Zhu Z., Wu Y., Luo J., Wang H. (2014). Preparation and the biopharmaceutical evaluation for the metered dose transdermal spray of dexketoprofen. *Journal of Drug Delivery*.

[166] Wang Y., Thakur R., Fan Q., Michniak B. (2005). Transdermal iontophoresis: combination strategies to improve transdermal iontophoretic drug delivery. *European Journal of Pharmaceutics and Biopharmaceutics*.

[167] Kim K. T., Lee J., Kim M. H. (2017). Novel reverse electrodialysis-driven iontophoretic system for topical and transdermal delivery of poorly permeable therapeutic agents. *Drug Delivery*.

[168] Park J., Lee H., Lim G. S., Kim N., Kim D., Kim Y. C. (2019). Enhanced transdermal drug delivery by sonophoresis and simultaneous application of sonophoresis and iontophoresis. *AAPS PharmSciTech*.

[169] Watanabe S., Takagi S., Ga K., Yamamoto K., Aoyagi T. (2009). Enhanced transdermal drug penetration by the simultaneous application of iontophoresis and sonophoresis. *Journal of Drug Delivery Science and Technology*.

[170] Le L., Kost J., Mitragotri S. (2000). Combined effect of low-frequency ultrasound and iontophoresis: applications for transdermal heparin delivery. *Pharmaceutical Research*.

[171] Hikima T., Ohsumi S., Shirouzu K., Tojo K. (2009). Mechanisms of synergistic skin penetration by sonophoresis and iontophoresis. *Biological and Pharmaceutical Bulletin*.

[172] Sawleshwarkar S. N., Salgaonkar V., Oberai C. M. (2003). Multicenter study to evaluate efficacy and irritation potential of benzoyl peroxide 4% cream in hydrophase base (Brevoxyl) in acne vulgaris. *Indian Journal of Dermatology Venereology and Leprology*.

[173] Weinberg J. M. (2006). The utility of benzoyl peroxide in hydrophase base (Brevoxyl) in the treatment of acne vulgaris. *Journal of Drugs in Dermatology*.

